# NEK7 Regulates NLRP3 Inflammasome Activation and Neuroinflammation Post-traumatic Brain Injury

**DOI:** 10.3389/fnmol.2019.00202

**Published:** 2019-08-29

**Authors:** Yuhua Chen, Jiao Meng, Fangfang Bi, Hua Li, Cuicui Chang, Chen Ji, Wei Liu

**Affiliations:** ^1^Department of Central Laboratory, Xi’an Peihua University, Xi’an, China; ^2^Department of Neurosurgery, the Second Affiliated Hospital of Xi’an Medical University, Xi’an, China; ^3^Department of Basic Medical Science Research Center, Shaanxi Fourth People’s Hospital, Xi’an, China

**Keywords:** traumatic brain injury, NLRP3 inflammasome, NEK7, caspase-1 activation, pyroptosis, potassium efflux, neuroinflammation

## Abstract

As one of the most common causes of mortality and disability, traumatic brain injury (TBI) is a huge psychological and economic burden to patients, families, and societies worldwide. Neuroinflammation reduction may be a favorable option to alleviate secondary brain injuries and ameliorate the outcome of TBI. The nucleotide-binding oligomerization domain, leucine-rich repeat and pyrin domain-containing 3 (NLRP3) inflammasome, has been shown to be involved in TBI. NIMA-related kinase 7 (NEK7) has been verified as an essential mediator of NLRP3 inflammasome activation that is recruited upstream of the formation of inflammasomes in response to NLRP3 activators. However, the underlying mechanism by which NEK7 operates post-TBI remains undefined. In this study, we performed both *in vivo* and *in vitro* experiments. Using an *in vivo* mouse TBI model, mice were administered an intracerebroventricular injection of NEK7-shRNA virus. For the *in vitro* analysis, primary cortical neurons with NEK7-shRNA were stimulated with lipopolysaccharide (LPS)/ATP or potassium (K^+^). We evaluated the effects of NEK7 knock-down on neurological deficits, NLRP3 inflammasomes, caspase-1 activation, and neuronal injury. During the 0–168 h post-TBI period *in vivo*, NEK7 and NLRP3 inflammasome activation increased in what appeared to be a time-dependent manner. As well as pyroptosis-related markers, caspase-1 activation (p20) and interleukin-1β (IL-1β) activation (p17) were up-regulated. NEK7 down-regulation attenuated neurological deficits, NLRP3 inflammasomes, caspase-1 activation, and neuronal injury. The same phenomena were observed during the *in vitro* experiments. Furthermore, NEK7 knock-down suppressed NLRP3 inflammasome activation and pyroptosis, which were triggered by K^+^ efflux, and the LPS + ATP-triggered NEK7–NLRP3 complex was reversed in primary cortical neurons placed in 50 mM K^+^ medium. Collectively, the data demonstrated that NEK7, as a modulator, regulates NLRP3 inflammasomes and downstream neuroinflammation in response to K^+^ efflux, through NEK7–NLRP3 assembly, pro-caspase-1 recruitment, caspase-1 activation, and pyroptosis in nerve injuries, post-TBI. NEK7 may be a potential therapeutic target for attenuating neuroinflammation and nerve injury post-TBI.

## Introduction

As one of the most common causes of mortality and disability, traumatic brain injury (TBI) causes a huge psychological and economic burden to patients, families, and societies worldwide (Barlow et al., 2018). The occurrence of TBI is an irreversible event, and includes both primary and secondary brain injuries. A series of complex pathophysiological processes lead to severe secondary brain injury within hours or days following acute brain injury (Maas et al., 2017). During the course of clinical treatment, primary brain injuries can progress to being untreatable in a short period of time, although the cascading pathology of secondary brain injuries that follow-on from the principal injury provides opportunities for intervention(Lazaridis et al., 2019).

Neuroinflammation is a major contributor to secondary brain injuries; therefore, its reduction may be a favorable option for alleviating secondary brain injuries and ameliorating the outcome of TBI (Cederberg and Siesjö, 2010; Mortezaee et al., 2018). Pyroptosis, a form of inflammatory cell death, features activation of caspase-1 and maturation of interleukin-1β (IL-1β) and IL-18 (Cederberg and Siesjö, 2010; Man et al., 2017; Ge et al., 2018). Caspase-1 is recognized as a pivotal mediating factor for pyroptosis. Caspase-1 cleaves gasdermin D (GSDMD) and the precursor of cytokines pro-IL-1β and pro-IL-18, which initiate pyroptosis and maturation of IL-1β and IL-18, respectively (Man et al., 2017); the N-terminal fragment of GSDMD also activates the nucleotide-binding oligomerization domain, leucine-rich repeat and pyrin domain-containing 3 (NLRP3) inflammasome, and caspase-1-dependent maturation of IL-1β and IL-18 (Man et al., 2017). Pyroptosis can be activated by NLRs or AIM2-like receptors through the triggering of inflammasomes, which contain apoptosis-associated speck-like protein containing CARD (ASC) and caspase-1, inducing a series of inflammatory reactions post-TBI (Ge et al., 2018; Guo et al., 2018). The subcutaneous injection of IL-1 receptor antagonists has been proven in clinical practice to ameliorate IL-1-mediated brain injury following TBI by regulating the neuroinflammatory reaction (Helmy et al., 2014, 2016). In our previous study, TBI was found to be accompanied by increased neuroinflammation and caspase-1-mediated pyroptosis in the acute phase post-TBI (Liu et al., 2018). Neuroinflammation, neurologic dysfunction, and nerve injury following TBI can be significantly improved by regulating the caspase-1-mediated pyroptosis signal pathway in neurons (Liu et al., 2018).

Recently, NLRP3 inflammasomes have been revealed to be involved in numerous neurological diseases (Johann et al., 2015; Saresella et al., 2016; Song et al., 2017); they also play a pivotal function during TBI (Mortezaee et al., 2018). Activated NLRP3 inflammasomes trigger caspase-1, causing the secretion of IL-1β and IL-18 and the initiation of pyroptosis (Zhou et al., 2018; Lazaridis et al., 2019). Emerging research has revealed that NIMA-related kinase 7 (NEK7), a Ser/Thr mitotic kinase, is a vital downstream mediator of potassium (K^+^) efflux, which is recruited to assist with the upstream formation of inflammasomes in response to NLRP3 activators (Schmid-Burgk et al., 2016). NEK7 is also detected in NLRP3/ASC complexes, and the formation of NLRP3 complexes that appear upstream of ASC complexes requires the interaction of NEK7 and NLRP3 (He et al., 2016). High (K^+^)_e_ can block the K^+^ efflux-induced interaction of NLRP3-NEK7 (He et al., 2016; Shi et al., 2016). However, the extent to which NEK7 regulates NLPRP3 inflammasomes in neurons is unknown.

To explore the mechanism of TBI, we performed an *in vivo* TBI mice model and *in vitro* cell model of inflammation and K^+^ stimulation. NEK7 shRNA was used as a tool to evaluate the role of NEK7 in downstream neuroinflammation post-TBI. Our results showed that down-regulated NEK7 reversed neurological deficits, NLRP3 inflammasome activation, and neuronal apoptosis. Ultimately, our findings suggest that NEK7 acts as a modulator, regulating NLRP3 inflammasomes and downstream inflammation in response to K^+^ efflux, through NEK7–NLRP3 assembly, pro-caspase-1 recruitment, caspase-1 activation, and pyroptosis in nerve injuries, post-TBI.

## Materials and Methods

### Controlled Cortical Impact (CCI) Model

Animal experiments were authorized by the Animal Care and Use Committee of Xi’an Peihua University, China. Male C57BL/6 mice were fed in the mouse-feeding facility with a 12-h/12-h light–dark cycle, with free foraging and activity.

The TBI model was established by a controlled cortical impact (CCI) device (Hatteras Instruments Inc., Cary, NC, USA). After anesthetized with 6% chloral hydrate (intraperitoneal injection), mice were placed on a brain stereotaxis instrument (Beijing Youchengjiaye Biotechnology Company, Limited, Beijing, China). The heads of the mice were wiped with 70% ethanol, the skin was cut at the midline, and a hole with 0.5 mm diameter in the right skulls, 2.0 mm posterior to the Bregma and 2.0 mm lateral to the sagittal suture, was drilled. The CCI device was used to induce the controllable TBI model with a general parameter (depth: 2 mm; velocity: 5.0 m/s; dwell time: 100 ms). After the injury, mice were immediately administered an intracerebroventricular injection of NEK7-shRNA virus or not. NEK7 (Locus ID 59125) Mouse shRNA (targeting sequence: CATTCTCGAAGAGTCATGCACAGAGATAT, Cat#TL515241, OriGene Technologies, Beijing, China) and a negative control (NC) pGFP-C-shLenti (Cat#TR30021V, OriGene Technologies) were prepared following the manufacturer’s instructions. The right lateral ventricles (depth: 3.0 mm) were injected with 2 μl (targeting NEK7 or NC) at 1 μl/min; the needle remained in place for 2 min and was slowly recovered once the injection was complete. Immediately after surgery, skull was sealed and the incision was sutured. The mice were placed on a heat pad until they regained consciousness and recovered gross locomotor function. After that, the mice were put back into normal feeding units and monitored. The sham group was subjected to normal surgical procedures but not CCI, and to virus *via* intracerebroventricular injection.

### Neurobehavioral Training and Evaluation

The experimenters were unaware of which mice received TBI or sham treatment. According to the previous reports (Liu et al., 2018), the modified neurological severity scoring (mNSS), Rotarod, and open-field tests were performed to evaluate neurological deficits.

### Water Content of Brain

As previously described (Kuriakose and Kanneganti, 2017; Chen et al., 2019), brain water content was assessed in 3-mm coronal sections of the ipsilateral cortex (or corresponding contralateral cortex), centered upon the impact site. Tissues were immediately weighed (wet weight) and then dehydrated at 100°C for 24 h to obtain the dry weight. Water content of the brain was calculated using the following formula: [(wet weight − dry weight)/wet weight] × 100.

### Injury Models of Primary Cortical Neurons

Primary cortical neurons were prepared as previously described (Liu et al., 2018). Briefly, cerebral cortices from embryonic C57BL/6 mice (day 18) were used for culture. Cortical tissue was obtained and digested using papayotin. Cells were collected after termination of digestion and cultured on poly-L-lysine-coated dishes with the DMEM medium, which was then replaced with the neuronal base medium (supplemented with 2% B27, 0.2 mM L-glutamine) overnight. The medium was changed every 3 days, and cells were used for subsequent experiments at 7–14 days.

Neurons were cultured for 10 days and then treated with lipopolysaccharide (LPS) or LPS + ATP: neurons were incubated with 10 ng/ml LPS (Sigma-Aldrich, St. Louis, MO, USA) for 4 h and 5 mM ATP (Sigma-Aldrich) for an additional 30 min. Cells or supernatant was then harvested for the next assays.

### NEK7 Knock-Down in Primary Cortical Neurons

NEK7 (Locus ID 59125) mouse shRNA (Cat#TL515241, OriGene Technologies) was used for NEK7 knock-down in primary cortical neurons, and pGFP-C-shLenti (Cat#TR30021V, OriGene Technologies) was used as an NC. NEK7 knock-down was performed following the manufacturer’s instructions. Knock-down efficiency was analyzed using a western blot (WB) analysis with anti-NEK7 antibody (Abcam, Cambridge, UK; ab133514).

### Incubation in K^+^-Free Medium and HIGH-K^+^ Medium

As previously described (Rühl and Broz, 2015), cells were treated with LPS and washed three times with the leaner (4.2 mM Na_2_CO_3_, 0.8 mM Na_2_HPO_4_, 1.3 mM CaCl_2_, 0.5 mM MgCl_2_, and 10 mM D-glucose monohydrate, pH 7.4) supplemented with 137 mM NaCl and 5 mM choline chloride. The control was washed with the leaner supplemented with 137 mM NaCl and 5 mM KCl. Cells were then incubated for 3 h in the respective buffers and then inflammasome activation and K^+^ concentrations were conducted.

### Stimulation in High-K^+^ Medium

According to Rühl and Broz (2015), cells were primed with LPS or LPS + ATP and washed three times with the leaner. On the basis of the leaner, the solutions with different K^+^ concentrations (5, 10, 20, and 40 mM) stimulated each group of cells. After incubation for 3 h, inflammasome activation and K^+^ concentrations were conducted.

### K^+^ Efflux Assay

Intracellular K^+^ detection was conducted as previously described (Rühl and Broz, 2015). Briefly, cells were added to 96-well plates and then lysed with ultrapure 3% HNO_3_ after stimulation. Intracellular K^+^ was then determined by inductively coupled plasma optical emission spectrometry using an Optima 2000 DV spectrometer (PerkinElmer Life Sciences, Waltham, MA, USA) using yttrium as an internal standard.

### Measurement of Cytokine and Lactate Dehydrogenase (LDH) Release

IL-1β, IL-18, and TNF-α were detected using the enzyme-linked immunosorbent assay (ELISA) kit (Anoric-Bio, Wuhan, China). Lactate dehydrogenase (LDH) was measured using a kit (Solarbio, Beijing, China). Syringe filters (0.2 μm) were used for filtering the supernatant to measure LDH secretion. The reaction mixture was added to the supernatant and incubated for 30 min. OD_450_ was measured using a microplate reader (Thermo Scientific, Waltham, MA, USA).

### TUNEL/NeuN and NLRP3/Caspase-1 Staining

TUNEL/NeuN stained as previously described (Chen et al., 2019), and brain tissues were obtained after transcardial perfusion, fixed with 4% paraformaldehyde, dehydrated, and embedded in paraffin. Paraffin coronal sections of thickness 4 μm (proximal to and located in the injury site) were stained using a DeadEnd™ fluorometric TUNEL system (Promega, Madison, WI, USA), followed by incubation overnight with primary anti-NeuN antibodies (Abcam, 1:400) and then incubated with secondary antibodies. Cell nuclei were stained with DAPI (Sigma-Aldrich). Images were obtained using a fluorescence microscope (Olympus, Osaka, Japan). NLRP3/caspase-1 staining was performed as the normal immunofluorescence operation, which was incubated with primary anti-NRLP3 antibodies (Abcam, 1:200) and caspase-1 antibodies (Abcam, 1:200).

### ASC Speck Staining

After fixation and permeabilization, the different treatment samples were blocked with 5% goat serum. Cells were incubated with anti-ASC antibody (#67824; CST, Danvers, MA, USA; 1:800) and secondary antibody (Alexa Fluor 594-conjugated, Abcam, 1:1,000). Nuclei were stained with DAPI. Images were captured using an inverted fluorescence microscope (Leica, Oskar-Barnack, Germany; IX71).

### WB Analysis

The total protein of cortical tissue proximal to or located in the injury site was lysed, and a BCA protein kit (Thermo Scientific) was used to quantify protein concentration. Following SDS-PAGE electrophoresis and western transfer, a PVDF membrane (Millipore, Billerica, MA, USA) was blocked with 5% BSA (Sigma-Aldrich) and incubated at 4°C overnight with the primary antibodies: NEK7 (Abcam, 1:1,000), NLRP3 (Abcam, 1:1,000), caspase-1 (Abcam, 1:1,000), caspase-1 (p20; AdipoGen, 1:1,000), ASC (CST, 1:1,200), GSDMD (Abcam, 1:1,000), IL-1β (R&D Systems, Minneapolis, MN, USA; 1:1,000), and GAPDH (CST, 1:10,000). Samples were then incubated with secondary antibodies (Abgent, 1:30,000), followed by chemiluminescent substrate development (Bio-Rad, Hercules, CA, USA), and detection using an imaging system (Bio-Rad).

Syringe filters (0.2 μm) were used for filtering the supernatant for the measurement of protein secretion, and Centricon Plus-20 centrifugal filter devices (Millipore) were used to concentrate the samples. Concentrated samples were separated with 15% SDS-PAGE and evaluated by WB analysis, as above.

The ASC oligomerization assay was performed as previously described (Shi et al., 2016). Cells were lysed using lysis buffer [0.5% Triton X-100, 150 mM NaCl, and 20 mM Hepes (pH 7.5)]. After centrifugation at 6,000 *g* at 4°C for 15 min, supernatant and pellets were used as the Triton-insoluble and Triton-soluble fractions, respectively. For the detection of ASC oligomerization, crude pellet was re-suspended in 300 μl of TBS buffer, and chemically cross-linked with 4 mM non-cleavable disuccinimidyl suberate (DSS) cross-linker for 30 min at 37°C and then centrifuged for 15 min at 6,000 *g*. After dissolving in SDS sample buffer, the samples were used for WB analysis.

### qRT-PCR Analysis

The total RNA of cortical tissue proximal to or located in the injury site was isolated using Trizol reagent (Invitrogen, Waltham, MA, USA). A HiFi-MMLV cDNA First Strand Synthesis Kit (CW Bio, Beijing, China) was used for reverse transcription. GoTaq qPCR Master Mix (Promega) was used for qRT-PCR analysis. Primer sequences were as follows: NEK7: F 5′-CCGTTACTCAGTTCCAGCCA-3′, R 5′-CTACCGGCACTCCATCCAAG-3′; NLRP3: F 5′-GCTAAGAAGGACCAGCCAGAGT-3′, R 5′-GAACCTGCTTCTCACATGTCGT-3′; ASC: F 5′-TGCTTAGAGACATGGGCTTAC-3′, R 5′-CTGTCCTTCAGTCAGCACACT-3′; caspase-1: F 5′-GACAAGGCACGGGACCTATGT-3′, R 5′-CAGTCAGTCCTGGAAATGTGC-3′; GAPDH: F 5′-AACTTTGGCATTGTGGAAGG-3′, R 5′-GGATGCAGGGATGATGTTCT-3′.

### Co-immunoprecipitation (CoIP)

Samples were lysed using a Pierce IP lysis buffer (Thermo Scientific) with proteinase inhibitor cocktail (Roche, Basel, Switzerland). After centrifugation with 4°C, 2,500 *g* for 5 min, suspensions were collected. Co-immunoprecipitation (CoIP) was performed using a Dynabeads™ CoIP Kit (Thermo Scientific), anti-NEK7 (Abcam, ab133514) or anti-NLRP3 (Abcam, ab214185) antibodies were coupled to dynabeads, and IgG served as the NC. Experimental operation was according to our previous study (Chen et al., 2019), and total proteins were mixed with antibody-coupled dynabeads and incubated overnight at 4°C. Adsorbed dynabeads were washed with wash buffer, and bound proteins were eluted with 20 μl of eluent, mixed with 20 μl of 2 × Laemmli buffer, and then boiled for 5 min. Finally, samples were loaded onto 4%–20% BeyoGel™ Plus PAGE (Beyotime, Shanghai, China) for electrophoresis.

### Statistical Analysis

Data are shown as means ± SEM. GraphPad Prism 7.0. software was used to perform the Student’s *t*-test or a one-way ANOVA followed by Tukey’s multiple comparison test. *P* < 0.05 was considered significant.

## Results

### Time Course of NEK7 and NLRP3 Inflammasome Post-TBI

**Figure 1:**
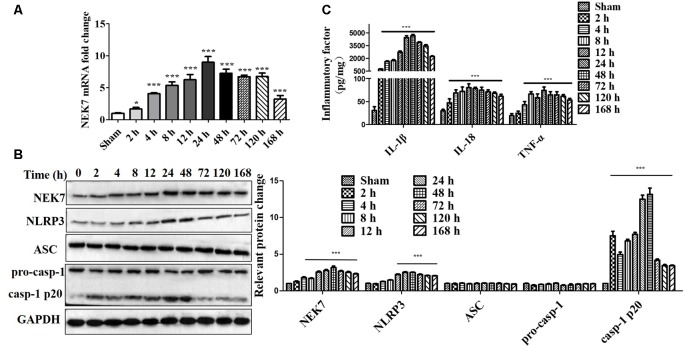
Time course of NIMA-related kinase 7 (NEK7) and nucleotide-binding oligomerization domain, leucine-rich repeat and pyrin domain-containing 3 (NLRP3) inflammasome in the acute phase post-traumatic brain injury (TBI). **(A)** Time course of NEK7 mRNA expression was analyzed during 168 h post-TBI, *n* = 10. **(B)** NEK7, NLRP3, apoptosis-associatedspeck-like protein containing CARD (ASC) and caspase-1 expression were detected during 168 h post-TBI, *n* = 6. **(C)** Time course of interleukin-1β (IL-1β), IL-18, and TNF-α levels during 168 h post-TBI, *n* = 10. Data are represented as the mean ± SEM. *p < 0.05, ***p < 0.001, vs. sham.

To evaluate whether NEK7 and NLRP3 inflammasomes were involved in TBI, the levels of NEK7 and NLRP3 inflammasomes in the peritraumatic cortex at different times were measured. As shown in Figures 1A,B, gene levels and protein expression were quantified at 2, 4, 8, 12, 24, 48, 72, 120, and 168 h post-TBI. NEK7 mRNA levels immediately increased within the first 2 h (*P* < 0.05) and reached the peak value at 24 h, and then remained at high levels until 168 h after TBI (*P* < 0.001). Levels of the proteins NEK7, NLRP3, and caspase-1 p20 were distinctly increased at 24 h; these high levels continued until 168 h post-TBI and following gradually decreased with time, and ASC and pro-caspase-1 levels appeared to increase slightly after TBI (Figure 1B). The IL-1β, IL-18, and TNF-α were analyzed (Figure 1C). IL-1β and IL-18 were immediately triggered at 2 h, TNF-α increased significantly by 4 h, and they all mildly decreased with time but still remained at high levels during 168 h post-TBI. These data indicate that NEK7 and NLRP3 inflammasomes show time dependence during the acute phase post-TBI.

### NEK7 Knock-Down Attenuates Neurological Deficits and Tissue Lesion After TBI

**Figure 2:**
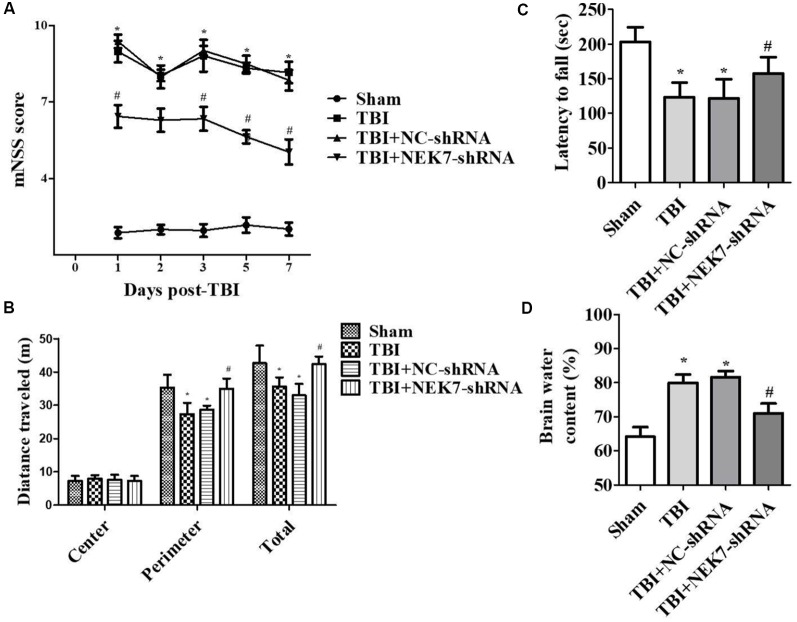
NEK7 down-regulation attenuates neurological deficits and cerebral edema post-TBI. Experimenters were unaware of the TBI or sham treatments with mice. Neurological performances were assessed by the modified neurological severity scoring (mNSS; **A**), the Rotarod test **(B)**, and the open-field test **(C)**. **(D)** The brain water content was detected following 48 h post-TBI. Data are represented as the means ± SEM, *n* = 11. *p < 0.05, vs. sham. ^#^*p* < 0.05, vs. TBI.

**Figure 3:**
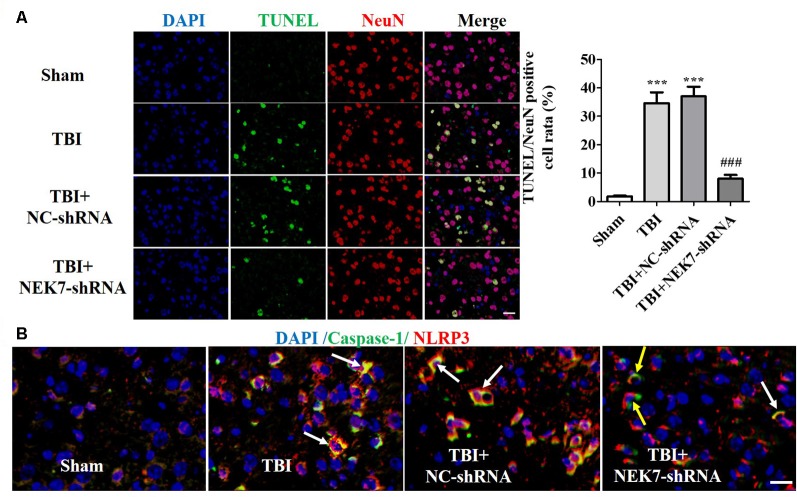
NEK7 down-regulation further reduces cortical neuronal damage. **(A)** Cortical injuries to coronal sections were conducted using TUNEL staining 48 h post-TBI. Nuclei were stained with DAPI and neurons were stained with NeuN. Arrows indicate significant neuronal damage. Histogram analysis of TUNEL/NeuN-positive cell rates. **(B)** NLRP3/caspase-1 stain was performed as the normal immunofluorescence operation. NEK7-shRNA suppressed the interaction with NLRP3 and caspase-1 after TBI. Scale bars: 20 μm. Data are represented as means ± SEM, *n* = 6. ***p < 0.001, vs. sham. ^###^*p* < 0.001, vs. TBI.

Prior to surgery, there were no differences in mNSS, Rotarod, or open-field scores in each group. In the mNSS test (Figure 2A), the sham group had low scores and showed no differences at any time point, whereas TBI induced significantly higher mNSS at 1 day followed by a gradual decline over time in the TBI group relative to the sham group (*P* < 0.05). The scores remained with no difference between the TBI group and the TBI + NC group post-TBI. The TBI + NEK7-shRNA group exhibited less mNSS compared with the TBI + NC group (*P* < 0.05). In the Rotarod test, the sham group showed the best performance compared with the TBI group at 48 h post-TBI (*P* < 0.05; Figure 2B). The scores were almost the same between the TBI group and the TBI + NC group. However, the scores were higher in the TBI + NEK7-shRNA group compared with the TBI + NC group (*P* < 0.05). In the open-field test, TBI mice moved less in the perimeter zone and less total distance (*P* < 0.05; Figure 2C). There were modest yet statistically significant differences in total distance traveled and perimeter zones between the TBI + NEK7-shRNA group and the TBI + NC group (*P* < 0.05). There was indifference between the TBI and TBI + NC groups. The brains of the mice were assessed post mortem. TBI caused a high water content of about 79.97%, while NEK7 knock-down distinctly inhibited edema of the brain (*P* < 0.05, vs. the TBI + NC group). However, there was indifference in edema between the TBI + NC group compared with the TBI group (Figure 2D). TUNEL staining was used to demonstrate cortical damage to coronal sections (Figure 3A). Lots of cortical neurons were stained with green fluorescence, revealing serious nerve damage post-TBI. TUNEL-positive neurons were reduced in NEK7-shRNA mice, and the TBI + NC group showed the same damage as the TBI group (Figure 3). As shown in Figure 3B, TBI induced the interaction with NLRP3 and caspase-1 and increased NLRP3/caspase-1 tagged cell, while NEK7-shRNA suppressed the interaction with NLRP3 and caspase-1 after TBI.

### NEK7 Knock-Down Blocks NLRP3 Inflammasome Activation After TBI

**Figure 4:**
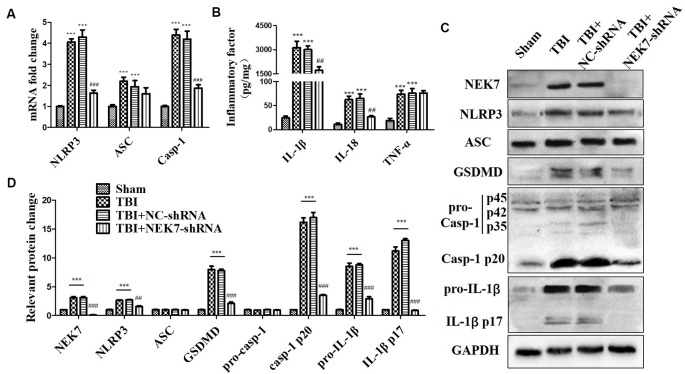
NEK7 knock-down suppresses NLRP3 inflammasome activation *in vivo*. At 48 h after TBI, qRT-PCR analysis of NLRP3, ASC, and caspase-1 mRNA level in cortical tissue **(A)**; enzyme-linked immunosorbent assay (ELISA) analysis of IL-1β, IL-18, and TNF-α level in cortex tissue **(B)**; and western blot (WB) analysis of NLRP3, ASC, gasdermin D (GSDMD), caspase-1, and IL-1β protein level in cortical tissue **(C)**. **(D)** Statistical analysis of NLRP3, ASC, GSDMD, caspase-1, and IL-1β protein level in cortical tissue. Data are represented as the means ± SEM, *n* = 8. ***p < 0.001, vs. sham. ^##^*p* < 0.01, ^###^*p* < 0.001, vs. TBI + negative control (NC).

As shown in the results above, TBI induced NLRP3 inflammasome activation and increased inflammation at 48 h post-TBI. The expression of NLRP3 inflammasome-related molecules were also detected in TBI + NC and TBI + NEK7-shRNA mice. Following NEK7 knock-down, NLRP3 and caspase-1 mRNA was significantly reduced (*P* < 0.001), but no significant alteration in ASC was observed (Figure 4A). NEK7 knock-down reversed the secretion of IL-1β and IL-18 (Figure 4B, *P* < 0.01), but TNF-α was unaffected in NEK7-shRNA mice (Figure 4B). Figures 4C,D showed that NLRP3, ASC, GSDMD, pro-caspase-1, caspase-1 p20, pro-IL-1β, and IL-1β p17 were suppressed in NEK7-shRNA mice post-TBI, with the reversals of GSDMD, caspase-1 p20, and IL-1β p17 being the most significant, compared with the TBI group (*P* < 0.001). This suggests that NEK7 down-regulation blocks NLRP3 inflammasome activation and its downstream activity.

### NEK7 Is Vital for NLRP3 Inflammasome Activation in Primary Cortical Neurons

**Figure 5:**
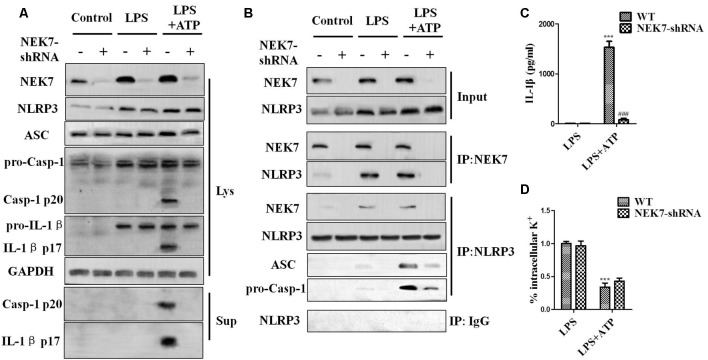
NEK7 knock-down attenuates NLRP3 inflammasome activation. Primary cortical neurons were transduced with NC-shRNA or NEK7-shRNA, and untreated or stimulated with lipopolysaccharide (LPS) or LPS + ATP. **(A)** Caspase-1 and IL-1β in the supernatant (Sup). and cell lysate (Lys). were analyzed in stimulated NC-shRNA- or NEK7-shRNA primary cortical neurons. **(B)** Co-immunoprecipitation (CoIP) analysis of the interaction between NEK7 and NLRP3 and the assembly of NLRP3 inflammasome. **(C)** IL-1β release was measured. **(D)** Analysis of intracellular potassium (K^+^) in each condition. Data are represented as the means ± SEM, *n* = 4. ***p < 0.001, vs. LPS. ^###^*p* < 0.001, vs. WT.

**Figure 6:**
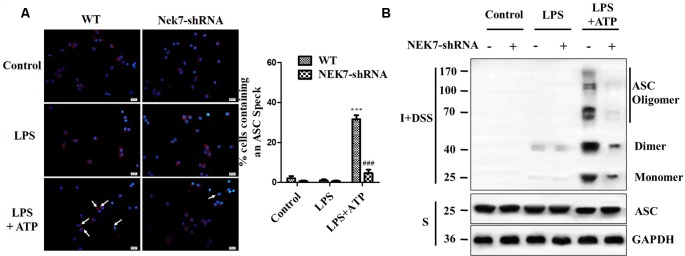
NEK7 is vital for NLRP3-mediated ASC speck formation in primary cortical neurons. **(A)** Immunofluorescence analysis of ASC specks (arrows) in neurons transfected with NC-shRNA or NEK7-shRNA. Neuros were untreated or stimulated with LPS or LPS + ATP. After treatments, cells were fixed and stained for ASC (red). Nuclear stained with DAPI (blue). Graphs show the means ± SEM, *n* = 4, more than 100 cells were counted in each test. **(B)** Cell lysates were solubilized with Triton X-100-containing buffer. Insoluble (I) fractions were cross-linked with disuccinimidyl suberate (DSS) to capture ASC oligomers (I+DSS). The soluble (S) and I+DSS fractions were analyzed by immunoblotting with ASC antibodies. ***p < 0.001, vs. control. ^###^*p* < 0.001, vs. WT.

When reconstituted with NEK7-shRNA, primary cortical neurons lacked NEK7 expression, but showed normal expression of NLRP3, caspase-1, and ASC (Figure 5A). LPS induced NLRP3, pro-caspase-1, and pro-IL-1β expression, but the activation of caspase-1 and IL-1β release was just triggered in LPS + ATP groups. NEK7-shRNA abolished the secretion of caspase-1 p20 and IL-1β p17 in LPS + ATP-induced neurons (Figure 5A). To demonstrate that NEK7 was critical for the assembly of NLRP3 inflammasomes, CoIP analysis was performed (Figure 5B). Interestingly, LPS alone induced weak interactions between NLRP3 and caspase-1 and ASC, but not caspase-1 activation, and that was consistent with the study of Shi et al. (2016). LPS + ATP triggered NLRP3 complexes, while NEK7 down-regulation reduced the interaction between NLRP3 and NEK7, suppressing the assembly of NLRP3 inflammasomes, including NLRP3–ASC complexes and pro-caspase-1 recruitment (Figure 5B). As shown in Figure 6, following neuronal stimulation by LPS + ATP, large spherical intracellular ASC speck was rapidly formed in the cytosol. However, the formation of ASC speck was suppressed in NEK7-shRNA neurons (Figure 6A). Also, a failure of ASC oligomerization was detected in NEK7-shRNA neurons compared to WT cells (Figure 6B). ELISA analysis showed that NEK7 down-regulation reversed LPS + ATP-induced secretion of IL-1β (Figure 5C). In addition, intracellular K^+^ levels were analyzed. Although LPS + ATP induced K^+^ efflux, NEK7 knock-down did not inhibit K^+^ efflux in primary cortical neurons (Figure 5D). The data indicate that NEK7 acts as a crucial role in conducting the inflammatory response, downstream of NLRP3 inflammasomes.

### NEK7 Is a Crucial Sensor of NLRP3 Activation Downstream of K^+^ Efflux in Primary Cortical Neurons

**Figure 7:**
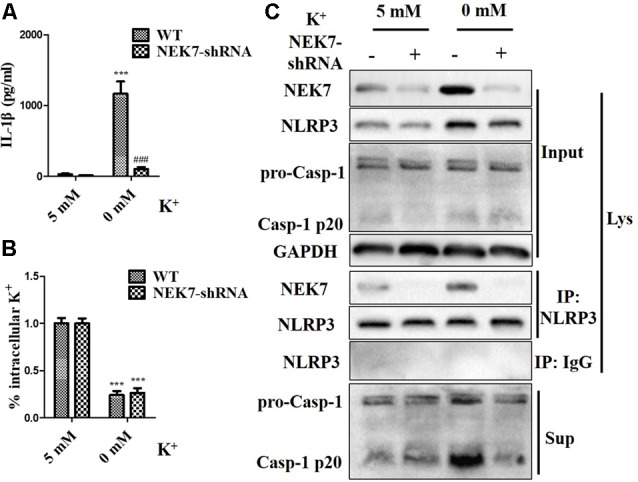
NEK7 knock-down alleviates NLRP3 inflammasome activation-induced K^+^-free medium. Primary cortical neurons were transduced with NC-shRNA or NEK7-shRNA, and treated with K^+^-free medium or normal medium. **(A)** Analysis of intracellular K^+^ in each condition. **(B)** IL-1β release was detected. **(C)** NEK7 and caspase-1 in the supernatant (Sup) and cell lysate (Lys) were analyzed. CoIP analysis of the interaction between NEK7 and NLRP3. Data are represented as the means ± SEM, *n* = 4. ***p < 0.001, vs. 5 mM group. ^###^*p* < 0.001, vs. WT.

**Figure 8:**
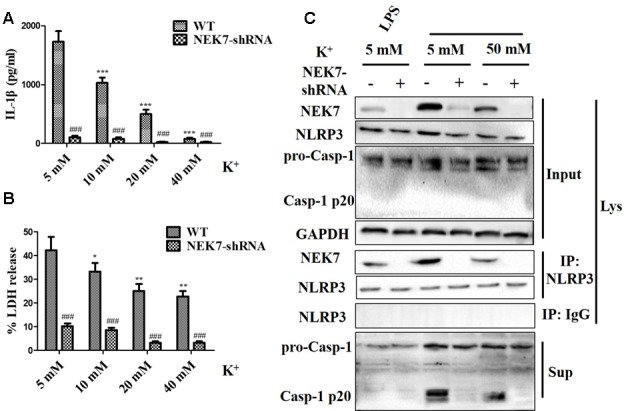
High-K^+^ medium alleviates NLRP3 inflammasome activation induced by LPS + ATP *via* NEK7. Primary cortical neurons were transduced with NC-shRNA or NEK7-shRNA, stimulated with LPS + ATP, and treated with different concentration high-K^+^ medium, then IL-1β **(A)** and lactate dehydrogenase (LDH; **B**) release were analyzed. **(C)** Cells were primed with LPS or LPS + ATP, and then stimulated with 50 mM high-K^+^ medium. NEK7 and caspase-1 in the supernatant (Sup) and cell lysate (Lys) were analyzed in stimulated NC-shRNA or NEK7-shRNA primary cortical neurons. CoIP analysis of the interaction between NEK7 and NLRP3. Data are represented as the means ± SEM, *n* = 4. *p < 0.05, **p < 0.01, ***p < 0.001, vs. 5 mM group. ^###^*p* < 0.001, vs. WT.

To verify that NEK7-mediated NLRP3 activation is downstream of K^+^ efflux, primary cortical neurons were transduced with NC-shRNA or NEK7 shRNA, stimulated with LPS or LPS + ATP, and then treated with K^+^-free medium or high-K^+^ medium. In NEK7-shRNA neurons, K^+^-free medium induced K^+^ efflux (Figure 7A) and IL-1β release (Figure 7B), while K^+^ efflux was unchanged and IL-1β release was reduced. Importantly, K^+^ efflux directly increased up-regulation of NEK7 expression and caspase-1 activation, including the plentiful releasing of caspase-1 p20 into the supernatant (Figure 7C); it also triggered the NLRP3 complex containing NEK7 (Figure 7C). K^+^ efflux did not influence the caspase-1 activation and NEK7–NLRP3 complexes in NEK7 knock-down cells (Figure 7C). It is well known that NEK7 expression is an event downstream of K^+^ efflux. Stimulation of primary cortical neurons with LPS + ATP induced comparable K^+^ efflux and treated with different concentration of high-K^+^ medium to resist K^+^ efflux. Notably, the release of IL-1β (Figure 8A) and LDH (Figure 8B) was blocked by the presence of high-K^+^ medium, which inhibited K^+^ efflux. NEK knock-down caused low levels of IL-1β and LDH to be released under LPS + ATP and high-K^+^ stimulation. LPS + ATP-initiated caspase-1 activation was eliminated by treatment with 50 mM KCl, which inhibited K^+^ efflux (Figure 8C). Moreover, NEK7 expression triggered by LPS + ATP was also reversed in 50 mM K^+^ medium (Figure 8C), and NLRP3–NEK7 complexes were not seen in neurons with 50 mM K^+^ medium (Figure 8C). The data suggest that K^+^ efflux triggers NEK7 and that this is a requirement for the assembly of NLRP3 inflammasomes and activation of caspase-1.

## Discussion

Currently, there is no approved drug with significant clinical efficacy for alleviating neuron damage following TBI. Several biomarkers of TBI, including UCH-L1, PNF-H, adenosine diphosphate, spermidine, and neurogranin, have been identified, but they carry the disadvantage of either not being sensitive or specific to TBI, which diminishes their clinical utility (Wang et al., 2018; Martinez and Stabenfeldt, 2019). The application of many emerging tools and techniques will improve the understanding of TBI and drive the exploration of many new candidate biomarkers for further characterization. Kerr et al. (2018) demonstrate that the inflammasome protein ASC may be used as a biomarker of TBI in serum and CSF, whereas caspase-1 may be used as a serum biomarker and IL-18 may be used as a biomarker in CSF. Increasingly, researches have identified that the acute phase after TBI is mainly associated with the production and release of a large number of cytokines release, such as the pro-inflammatory mediators TNF, IFN-γ, IL-1β, and IL-6, as well as the anti-inflammatory cytokines IL-4 and IL-10 (Jassam et al., 2017; Webster et al., 2017). IL-1β, IL-18, and other inflammatory cytokines trigger secondary inflammatory cascades in the central nervous system (CNS), sustaining neuroinflammation and releasing neurotoxic inflammatory mediators (Freeman and Ting, 2016). Inflammasomes have been involved in the pathogenesis of multiple CNS diseases, such as Alzheimer’s disease, epilepsy, and TBI (Mamik and Power, 2017). The neuroinflammatory cascade includes a wide array of both humoral and cellular players post-TBI. In some ways, this neuroinflammation modulation provides various potential opportunities for therapeutic intervention. Despite this, the neuroinflammatory process in TBI has not been recognized as a gold standard for evaluation. Measuring the concentration of biomarkers, such as chemokines and cytokines, during sustained neuroinflammation continues to be the most common method used in clinical studies. Some clinical studies showed that IL-6, IL-1β, and IL-8 occurred within the first 48–72 h following TBI, with IL-10 remaining at high levels throughout the acute phase for up to 5 days of monitoring in patients with severe TBI (Thelin et al., 2017; Zeiler et al., 2017). Additionally, the subcutaneous injection of recombinant human IL-1 receptor antagonists, with the aim of alleviating IL-1-mediated brain injury by regulating the neuroinflammatory reaction following TBI, has been proven in clinical practice (Helmy et al., 2014, 2016). Our current findings demonstrated that NEK7 knock-down modulated ASC oligomerization, caspase-1 activation, and neuroinflammatory factors, such as maturation and secretion of IL-1β and IL-18, and significantly reduced inflammation and nerve damage post-TBI.

Inflammasome activation and pyroptosis have been verified in multiple CNS cell types (Walsh et al., 2014). As a caspase-1-dependent inflammatory necrosis, pyroptosis can be induced by a range of microbial infections as well as other, non-infectious causes, such as TBI. NLRP3, NLRC4, AIM2, and Pyrin inflammasomes activation are involved in initiating pyroptosis, and maturation of IL-1β and NLRP3 inflammasome is responsible for caspase-1 activation (Man and Kanneganti, 2015; Yuan et al., 2016). Some studies have confirmed that AIM2 and NLRs inflammasomes mediate caspase-1 activation and neuronal pyroptosis during CNS infection and injury, including TBI (Ge et al., 2018; Guo et al., 2018; Mortezaee et al., 2018), and Adamczak et al. (2014) have demonstrated that CSF from TBI patients can activate AIM2-mediated pyroptosis in neurons. Caspase-1 deficiency or its inhibitors exhibit protection in experimental stroke models, as do oxygen and glucose deprivation in rat organotypic hippocampal tissue (Ray et al., 2000; Ross et al., 2007). Caspase-1 suppression obliterates inflammasome activation and pyroptosis of glia in multiple sclerosis models (McKenzie et al., 2018). In experimental animal models of autoimmune encephalomyelitis, it was shown that treatment with the caspase-1-specific inhibitor, VX-765, decreased the levels of inflammasome and pyroptosis-associated proteins (IL-1β, IL-18, and GSDMD) in the CNS, prevented axonal injuries, and improved neurobehavioral performance (McKenzie et al., 2018). In our previous studies, TBI in mice was found to be accompanied by multiple neuroinflammation and caspase-1-mediated pyroptosis effects during the acute phase, while the neuroinflammatory, neurologic dysfunction, and nerve injury following TBI were shown to be significantly improved by regulating the pyroptosis signal pathway *via* caspase-1 inhibition (Liu et al., 2018). We also demonstrated that NEK7 knock-down reduced caspase-1 activation, including p20 fragments, resulting in the inhibition of pyroptosis and downstream inflammation after TBI.

As a key regulatory molecule upstream of caspase-1 activation and pyroptosis, NLRP3 has been verified to play a vital role in TBI. NLRP3 is a broad mediator of cellular environmental homeostasis and triggered downstream of excessive stimuli; NLRP3 then assembles a multiprotein platform resulting in caspase-1 activation, that controls, by direct cleavage, the maturation of cytosolic pro-cytokines containing pro-interleukin-1β (Groslambert and Py, 2018). Furthermore, caspase-1 processes cytosolic GSDMD and liberates its pore-forming N-terminal domain, causing the release of abundant mature cytokines, as well as pyroptotic cell lysis (Groslambert and Py, 2018). Considerable research has shown that increased NLRP3 levels are detected in the CSF of patients with severe TBI, and NLRP3 inflammasome complexes, including ASC and caspase-1, have also been discovered in the cerebral cortex of TBI animal models (Wallisch et al., 2017), suggesting that NLRP3 can be regarded as a prognostic indicator for TBI (Qian et al., 2017). Recently, research has found that MCC950, a selective inhibitor of NLRP3 inflammasomes, provides a significant reduction in neurological dysfunction, neuroinflammation, cerebral edema, traumatic area, and cell death, through the suppression of microglial activation and pro-inflammatory cytokine release in animals with TBI (Ismael et al., 2018; Xu K. Y. et al., 2018). Irrera et al. (2017) demonstrate that NLRP3 deficiency also improves recovery in mice following TBI. Notably, NLRP3 repression may be a potential therapeutic strategy for patients with TBI. We demonstrated that NEK7 down-regulation attenuated NEK7–NLRP3 complexes, ASC specks, and NLRP3 inflammasome activation, to suppress pro-inflammatory cytokine release following TBI.

A recent study has confirmed that the NEK7–NLRP3 signal pathway appears to have clinical significance in systemic lupus erythematosus patients and is inversely correlated with disease activity (Ma et al., 2018). As a highly conserved serine/threonine kinase, NEK7 is located at centrosomes and is necessary for the progression of both mitosis and the cell cycle. In fact, it is pivotal for mitosis, which is related to cell division and growth. The normal activity of NEK7 is a requirement for dendritic growth and branching, as well as the correct morphology of neurons. NEK7 conducts the processes in part *via* phosphorylation of Eg5/KIF11, accelerating its accumulation on microtubules in distal dendrites (Freixo et al., 2018). NEK7 is involved in microtubule polymerization and mainly expresses in parvalbumin (PV+) interneurons at the time when cells establish their connectivity. NEK7-deficient PV+ interneurons appear as alterations in microtubule dynamics, axon growth-cone steering, arbor complexity, and total number of synaptic contacts formed with pyramidal cells, as well as decreased axon length (Hinojosa et al., 2018). NEK7 activity is lower in cells under normal growth conditions (Minoguchi et al., 2003). However, numerous basic studies have confirmed that NEK7 is upregulated when stimulated by serum depletion (in NIH3T3 cells; Minoguchi et al., 2003) and external inflammation (in macrophages and damaged lung tissue; Green et al., 2018; Xu X. et al., 2018). Up-regulated NEK7 triggers the production of abnormal cells, including polykaryocytes and apoptotic cells that are related with inflammation (Xu et al., 2016). Researches have indicated that NEK7 targets NLRP3, and pinpointing the precise role of NEK7 in inducing NLRP3 inflammasomes can provide the basis for NEK7 to be the ultimate target in remedying inflammatory diseases linked to NLRP3 inflammasomes (He et al., 2016). According to Shi et al. (2016), NEK7 can be coimmunoprecipitated with NLRP3, but not with NLRC4 or AIM2. NEK7 bridges adjacent NLRP3 subunits with bipartite interactions to mediate the activation of the NLRP3 inflammasome (Sharif et al., 2019). We demonstrated that TBI induces NEK7 up-regulation and that NEK7 knock-down is beneficial for suppressing NLRP3 inflammasome activation and nerve injury. This may be contrary to the effect NEK7 has for maintaining dendritic morphogenesis in neurons. NEK7 exhibits a low level of activity under normal circumstances and is pivotal for maintaining homeostasis. If external stimuli lead to an imbalance of NEK7, corresponding negative events may occur. As Shi et al. reported (Shi et al., 2016), NEK7 performs non-redundant functions in NLRP3 inflammasome activation and mitosis that cannot be synchronized. They showed that caspase-1 activation and IL-1β production triggered by LPS + ATP were enhanced during interphase but greatly reduced during mitosis, NEK7 overexpression partially rescued IL-1β production, and LPS + ATP stimulation failed to induce endogenous NEK7–NLRP3 interaction in mitotic peritoneal macrophages (Shi et al., 2016). During mitosis, actin and microtubule cytoskeleton components undergo dramatic restructuring, and transcription and translation slow or stop. NLRP3-dependent inflammatory responses may be abolished during these cellular changes, since they require microtubule polymerization, localization of NLRP3 and ASC at the perinuclear region, secretion of IL-1β and IL-18, and the triggering of IL-1β- and IL-18-dependent transcriptional programs (Eder, 2009; Shi et al., 2016). NEK7 dependence may have evolved as a switch to avert an invalid or potentially destructive inflammatory response during mitosis. We have shown that external stimuli can induce NEK7-mediated NLRP3 inflammasome activation, including NLRP3 inflammasome assembly, ASC speck formation (ASC oligomerization), caspase-1 activation, and IL-1β production, and that NEK7 knock-down can reverse a series of inflammatory reactions, both *in vivo* and *in vitro*. Furthermore, we treated primary cortical neurons with shRNA-NC or NEK7 shRNA, stimulated them with LPS or LPS + ATP, and incubated them with K^+^-free medium or high-K^+^ medium. Although LPS does not adequately mimic the injury process occurring as a result of TBI, LPS was used in this study to prime the inflammasome for activation. Thus, NEK7 is shown to be a primary sensor of NLRP3 activation downstream of K^+^ efflux in primary cortical neurons.

Taken together, NEK7 is a specific and vital factor, which possesses non-redundant functions downstream of K^+^ efflux binding to NLRP3 and is a requirement for inflammasome assembly. This suggests that hyperactive NLRP3 inflammasomes may result from an increased association between NEK7 and NLRP3 following TBI. NEK7 knock-down drives depressed NEK7–NLRP3 to alleviate some of the effects of caspase-1 activation, pyroptosis, neuroinflammation, neuronal damage, and neurological deficits, which are all involved in the acute phase post-TBI. Further work to illuminate the dual roles of NEK7 is necessary. Thus, these findings indicate that NEK7 may be a potential therapeutic target to attenuate neuroinflammation and nerve injury post-TBI.

## Data Availability

The datasets generated for this study are available on request to the corresponding author.

## Ethics Statement

### Animal Subjects

The animal study was reviewed and approved by The Animal Care and Use Committee of Xi’an Peihua University.

## Author Contributions

YC and WL designed the work. JM and FB developed methodology. CC and CJ carried out the experiments. YC, JM, and FB performed the data analysis and created the figures. YC wrote the first draft. WL and HL reviewed and revised the manuscript, and supervised the study. All authors read and approved the manuscript.

## Conflict of Interest Statement

The authors declare that the research was conducted in the absence of any commercial or financial relationships that could be construed as a potential conflict of interest.

## Abbreviations

TBI: traumatic brain injury
NLRP3: nucleotide-binding oligomerization domain, leucine-rich repeat and pyrin domain-containing 3
NEK7: NIMA-related kinase 7
K^+^: potassium
ASC: apoptosis-associated speck-like protein containing CARD
NLRs: NOD-like receptors
mNSS: modified neurological severity scoring
LPS: lipopolysaccharide
WB: Western blot
CoIP: co-immunoprecipitation.
