# Corrigendum: NEK7 Regulates NLRP3 Inflammasome Activation and Neuroinflammation Post-traumatic Brain Injury

**DOI:** 10.3389/fnmol.2019.00247

**Published:** 2019-10-15

**Authors:** Yuhua Chen, Jiao Meng, Fangfang Bi, Hua Li, Cuicui Chang, Chen Ji, Wei Liu

**Affiliations:** ^1^Department of Central Laboratory, Xi'an Peihua University, Xi'an, China; ^2^Department of Neurosurgery, the Second Affiliated Hospital of Xi'an Medical University, Xi'an, China; ^3^Department of Basic Medical Science Research Center, Shaanxi Fourth People's Hospital, Xi'an, China

**Keywords:** traumatic brain injury, NLRP3 inflammasome, NEK7, caspase-1 activation, pyroptosis, potassium efflux, neuroinflammation

In the original article, there was an error. The title is missing a letter “N” at the beginning. “EK7 Regulates NLRP3 Inflammasome Activation and Neuroinflammation Post-traumatic Brain Injury.”

A correction has been made to the ***Title***.

**NEK7 Regulates NLRP3 Inflammasome Activation and Neuroinflammation Post-traumatic Brain Injury**.

The authors apologize for this error and state that this does not change the scientific conclusions of the article in any way. The original article has been updated.

